# Draft genome sequences of two *Gluconobacter* strains isolated from spoiled orange juice

**DOI:** 10.1128/mra.00489-24

**Published:** 2024-07-22

**Authors:** Ky Ariano, Tayler Farrington, Paul Schweiger

**Affiliations:** 1 Department of Microbiology, University of Wisconsin - La Crosse, La Crosse, Wisconsin, USA; The University of Arizona, Tucson, Arizona, USA

**Keywords:** acetic acid bacteria, genome sequencing, environmental microbiology, genomics, whole genome

## Abstract

Here, we present the draft genome sequences of two *Gluconobacter* strains that were isolated from spoiled orange juice. *Gluconobacter* are members of the acetic acid bacteria and are known for their unique metabolism and use in industry. Understanding acetic acid bacteria diversity is essential for engineering further optimized industrial strains.

## ANNOUNCEMENT

Acetic acid bacteria (AAB) incompletely oxidize growth substrates using membrane-bound dehydrogenases (1, 2). The byproducts diffuse into the media, which makes them industrially useful for production of vinegar, vitamin C, the antidiabetic drug miglitol, dihydroxyacetone, and many artificial flavorings (3 – 5). To expand upon known AAB diversity and identify new targets for strain design, we sequenced the genomes of two *Gluconobacter* strains.

These *Gluconobacter* strains were isolated from spoiled Fit & Active Orange Juice Beverage purchased in Onalaska, WI. Samples were plated onto YG medium (6 g/L yeast extract, 20 g/L glucose, 2.5 g/L MgSO_4_ × 7H_2_O, 1 g/L (NH_4_)_2_SO_4_, 1 g/L KH_2_PO_4_, pH 6.0) with 50 µg/mL cefoxitin and incubated at 30°C for 3 days. For initial isolation, 20 g/L CaCO_3_ was added to differentiate AAB colonies, and 15 g/L agar was added to make solid media. Isolated colonies producing zones of clearance were selected and passaged to purity. Genomic DNA was isolated from single colonies grown in YG broth using the DNeasy UltraClean Microbial Kit (QIAGEN, Ann Arbor, MI). The 16S rRNA gene was amplified using primers 8FE/1492R and Sanger sequenced by Eurofins Genomics (Louisville, KY) using their 16F primer (CGGTTACCTTGTTACGACTT). Two isolates were identified as *Gluconobacter* spp. using the NCBI BLAST RefSeq database. The isolates *Gluconobacter* sp. OJA and OJB best matched *G. cerinus* (99.90% identity, *E*-value: 0.0, accession: NR_118192) and *G. cerevisiae* (99.88% identity, *E*-value: 0.0, accession: NR_117735).

For whole-genome sequencing, *Gluconobacter* sp. OJA and OJB were grown on YG agar from 15% glycerol stocks stored at −80°C. Single colonies were inoculated into YG broth with 50 µg/mL cefoxitin at 30°C with 250 rpm shaking overnight. Genomic DNA was isolated as described above. DNA was quantified using the QuantiFlor dsDNA System (Promega, Madison, Wisconsin) and sent to SeqCenter (Pittsburgh, PA) for sequencing. Illumina libraries were prepared using an Illumina DNA Prep Kit and IDT 10 bp UDI indices and sequenced on a NextSeq 2000 producing 2 × 151 bp reads. Demultiplexing and adapter removal used bcl2fastq v.2.20.0.422. Nanopore sample preparation used the Oxford Nanopore Genomic DNA by Ligation kit (SQK-LSK109) and protocol without shearing. Samples were run on a MinION using Nanopore R9 flow cells (R9.4.1). Quality control and basecalling used Guppy v5.0.16 in high-accuracy base mode (6).

Reads were processed and assembled using KBase and the Bacterial and Viral Bioinformatics Resource Center (7, 8). All software used default parameters except where noted. Read libraries were quality checked using FastQC v0.12.1 and Compute Simple Read Library Stats v2.0.2. Low-quality reads and adapter sequences were trimmed using the JGI RQCFilter pipeline (BBTools v38.22) v0.5.0, Trim Galore v0.6.5dev, and CutAdapt v4.2 (9). Trimmed reads were assembled using HybridSPAdes v3.15.3 (minimum contig length: 2,000 bp, K-mer sizes: 21, 33, 55, 77, 99, and 127). The assembled genome was annotated using the NCBI Prokaryotic Genome Annotation Pipeline (PGAP) v6.7 and deposited to NCBI (10). Summary statistics and relevant reporting information are given in Table 1.

**TABLE 1:** Genome assembly statistics, annotation features, and data accession numbers

Strain	*Gluconobacter* sp. OJA	*Gluconobacter* sp. OJB
Illumina data		
No. of raw reads generated	5,519,762	6,368,484
No. of raw Mb generated	811.69	936.36
No. of reads after filtering	5,295,054	6,109,328
No. of Mb after filtering	733.67	892.74
Fold coverage	211.83	280.65
SRA ID	SRX24435052	SRX24435054
Nanopore data		
No. of raw reads generated	105,690	539,216
No. of raw Mb generated	490.66	2137.17
*N*_50_	11,732	7,350
No. of reads after filtering	105,690	539,216
No. of Mb after filtering	401.46	1835.80
Fold coverage	121.80	624.20
SRA ID	SRX24435053	SRX24435055
Hybrid assembly		
Total length	3,794,501	3,305,620
*N*_50_	2,287,605	2,819,765
*L*_50_	1	1
No. of contigs	11	10
GC content (%)	56.19	58.21
Completeness (%)^*a*^	97.93	96.01
Contamination (%)^*a*^	7.44	7.58
Genome annotation		
Protein-coding genes	3,419	3,054
5S rRNA	4	4
16S rRNA	4	4
23S rRNA	4	4
tRNAs	57	58
Taxonomic classification		
Placement reference	GCA_002723935.1	GCA_018811965.1
Placement taxonomy^*b*^	*Gluconobacter cerinus*	*Gluconobacter cerevisiae*
Placement ANI	96.95	96.45
Accession numbers		
BioProject accession	PRJNA1107211	PRJNA1107211
Biosample accession	SAMN41163485	SAMN41163486
GenBank accession	JBDIWI000000000	JBDIWH000000000

^*a*^ Predicted with CheckM v1.2.2 within the PGAP v6.7.

^*b*^ Taxonomic assignment performed using the PGAP v6.7.

## Data Availability

The draft genome sequences of *Gluconobacter* sp. OJA and *Gluconobacter* sp. OJB have been deposited at NCBI. Their accession numbers are listed in Table 1.

## References

[1] Deppenmeier U , Ehrenreich A . 2009. Physiology of acetic acid bacteria in light of the genome sequence of Gluconobacter oxydans. J Mol Microbiol Biotechnol 16:69–80. doi:10.1159/000142895 18957863

[2] Prust C , Hoffmeister M , Liesegang H , Wiezer A , Fricke WF , Ehrenreich A , Gottschalk G , Deppenmeier U . 2005. Complete genome sequence of the acetic acid bacterium Gluconobacter oxydans. Nat Biotechnol 23:195–200. doi:10.1038/nbt1062 15665824

[3] Deppenmeier U , Hoffmeister M , Prust C . 2002. Biochemistry and biotechnological applications of Gluconobacter strains. Appl Microbiol Biotechnol 60:233–242. doi:10.1007/s00253-002-1114-5 12436304

[4] da Silva GAR , Oliveira S de S , Lima SF , do Nascimento RP , Baptista A de S , Fiaux SB . 2022. The industrial versatility of Gluconobacter oxydans: current applications and future perspectives. World J Microbiol Biotechnol 38:134. doi:10.1007/s11274-022-03310-8 35688964 PMC9187504

[5] Wang EX , Ding MZ , Ma Q , Dong XT , Yuan YJ . 2016. Reorganization of a synthetic microbial consortium for one-step vitamin C fermentation. Microb Cell Fact 15:21. doi:10.1186/s12934-016-0418-6 26809519 PMC4727326

[6] Sherathiya VN , Schaid MD , Seiler JL , Lopez GC , Lerner TN . 2021. GuPPy, a Python toolbox for the analysis of fiber photometry data. Sci Rep 11:24212. doi:10.1038/s41598-021-03626-9 34930955 PMC8688475

[7] Arkin AP , Cottingham RW , Henry CS , Harris NL , Stevens RL , Maslov S , Dehal P , Ware D , Perez F , Canon S , et al. . 2018. KBase: the United States department of energy systems biology knowledgebase. Nat Biotechnol 36:566–569. doi:10.1038/nbt.4163 29979655 PMC6870991

[8] Olson RD , Assaf R , Brettin T , Conrad N , Cucinell C , Davis JJ , Dempsey DM , Dickerman A , Dietrich EM , Kenyon RW , et al. . 2023. Introducing the bacterial and viral bioinformatics resource center (BV-BRC): a resource combining PATRIC, IRD and ViPR. Nucleic Acids Res 51:D678–D689. doi:10.1093/nar/gkac1003 36350631 PMC9825582

[9] Martin M . 2011. Cutadapt removes adapter sequences from high-throughput sequencing reads. EMBnet J 17:10. doi:10.14806/ej.17.1.200

[10] Tatusova T , DiCuccio M , Badretdin A , Chetvernin V , Nawrocki EP , Zaslavsky L , Lomsadze A , Pruitt KD , Borodovsky M , Ostell J . 2016. NCBI prokaryotic genome annotation pipeline. Nucleic Acids Res 44:6614–6624. doi:10.1093/nar/gkw569 27342282 PMC5001611

